# Caution needs to be taken when assigning transcription start sites to ends of protein-coding genes: a rebuttal

**DOI:** 10.1186/s40246-018-0164-4

**Published:** 2018-06-27

**Authors:** Niv Sabath, Anna Vilborg, Joan A. Steitz, Reut Shalgi

**Affiliations:** 1 Department of Biochemistry, Rappaport Faculty of Medicine, Technion—Israel Institute of Technology, 31096 Haifa, Israel; 2 Department of Molecular Biophysics and Biochemistry, Howard Hughes Medical Institute, Boyer Center for Molecular Medicine, Yale University School of Medicine, 295 Congress Avenue, New Haven, CT 06536 USA

**Keywords:** TSS-Seq, Cap-Seq, snoRNAs

## Abstract

Naturally occurring stress-induced transcriptional readthrough is a recently discovered phenomenon, in which stress conditions lead to dramatic induction of long transcripts as a result of transcription termination failure. In 2015, we reported the induction of such *d*ownstream *o*f *g*ene (DoG) containing transcripts upon osmotic stress in human cells, while others observed similar transcripts in virus-infected and cancer cells. Using the rigorous methodology Cap-Seq, we demonstrated that DoGs result from transcriptional readthrough, not de novo initiation. More recently, we presented a genome-wide comparison of NIH3T3 mouse cells subjected to osmotic, heat, and oxidative stress and concluded that massive induction of transcriptional readthrough is a hallmark of the mammalian stress response. In their recent letter, Huang and Liu in contrast claim that DoG transcripts result from novel transcription initiation near the ends of genes. Their conclusions rest on analyses of a publicly available transcription start site (TSS-Seq) dataset from unstressed NIH3T3 cells. Here, we present evidence that this dataset identifies not only true transcription start sites, TSSs, but also 5′-ends of numerous snoRNAs, which are generally processed from introns in mammalian cells. We show that failure to recognize these erroneous assignments in the TSS-Seq dataset, as well as ignoring published Cap-Seq data on TSS mapping during osmotic stress, have led to misinterpretation by Huang and Liu. We conclude that, contrary to the claims made by Huang and Liu, TSS-Seq reads near gene ends cannot explain the existence of DoGs, nor their stress-mediated induction. Rather it is, as we originally demonstrated, transcriptional readthrough that leads to the formation of DoGs.

## Background

In 2015, we reported the induction of *d*ownstream *o*f *g*ene (DoG) containing transcripts upon osmotic stress in human cells [1]. Other labs have observed similar transcripts following viral infection, in renal cancer, and more [2–4]. We used the rigorous methodology Cap-Seq [5], before and after subjecting human cells to osmotic stress, to capture transcription start sites (TSSs) in a genome-wide manner, in order to ask whether stress-induced DoGs are independent transcripts or rather continuous with their upstream gene, i.e., a product of transcriptional readthrough. Our data demonstrated that DoGs result from transcriptional readthrough, not de novo initiation. More recently, we presented a genome-wide comparison of NIH3T3 mouse cells subjected to osmotic, heat, and oxidative stress and concluded that massive induction of transcriptional readthrough is a hallmark of the mammalian stress response [6].

In their recent letter, Huang and Liu [7], in contrast, claim that DoG transcripts result from novel transcription initiation near the ends of genes. Their conclusions rest on the analysis of a publicly available transcription start site (TSS-Seq) dataset from unstressed NIH3T3 cells [8]. Here, we present evidence that this dataset identifies not only true TSSs but also 5′-ends of numerous snoRNAs, usually processed from introns. Neglecting to discard these highly abundant contaminants, as well as failure to carry out additional necessary controls, have led Huang and Liu to draw erroneous conclusions. We demonstrate here that, contrary to Huang and Liu’s assertion, TSS-Seq peaks near gene ends cannot explain the existence of DoGs, nor can it explain their stress-mediated induction.

## TSS-Seq data efficiently capture snoRNAs, which originate from transcript introns

**Fig. 1:**
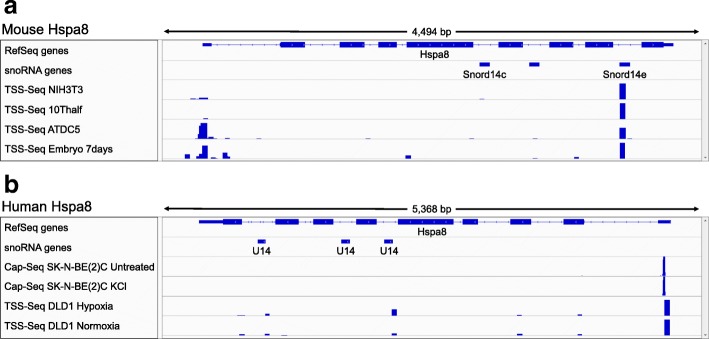
The TSS-Seq peak near the 3′-end of Hspa8 originates from a snoRNA. **a** An IGV plot shows TSS-Seq data from four mouse cell lines [8] at the Hspa8 locus. snoRNA genes are indicated. **b** An IGV plot shows Cap-Seq data for unstressed and osmotic (KCl) stressed human SK-N-BE(2)C cells from [1] and TSS-Seq data from human DLD1 cells [8] at the Hspa8 locus. snoRNA genes are indicated

In their recent letter [7], Huang and Liu reported that one pan-stress DoG-producing gene out of more than 1800 found in our recent study [6], Hspa8, exhibits a high TSS-Seq peak near its 3′-end. They concluded that the Hspa8 DoG, doHspa8, is not a readthrough transcript but a lncRNA with an independent promoter. Close examination of the same data reveals that this TSS-Seq peak marks the exact 5′-end of the snoRNA Snord14e (Fig. 1a) within the last intron of Hspa8. Huang and Liu noted the association between the TSS-Seq peak and Snord14e, but rather interpreted it as evidence of a novel lncRNA. As it is well known that snoRNAs are processed from introns [9], it is highly unlikely that these TSS-Seq peaks correspond to the start of lncRNAs.

We then looked for other snoRNAs in the NIH3T3 TSS-Seq dataset and uncovered more than 1000 snoRNAs with TSS-Seq reads genome-wide, of which 249 had significant peaks (> 50). Thus, it is evident that the TSS-Seq method captures highly structured snoRNAs quite efficiently. We further examined additional TSS-Seq datasets from DBTSS [8] of human and mouse cell lines. We identified a significant TSS-Seq peak for Snord14e for all mouse samples examined (Fig. 1a) and a lower level peak in the human DLD1 samples within the Hspa8 gene (Fig. 1b).

The high abundance of snoRNA reads in TSS-Seq data might be the result of incomplete removal of 5′-phosphates by alkaline phosphatase, an early step in the preparation of RNA for analysis [8], which can be ascribed to the tight RNA secondary structure at the 5′-ends of snoRNAs. In contrast, the Cap-Seq protocol relies on the presence of a 5′-m7G cap in addition to the removal of 5′-phosphates, which makes it significantly more rigorous in identifying true TSSs [5]; indeed, it does not capture snoRNA 5′-ends (Fig. 1b).

Therefore, these analyses demonstrate that caution is advisable when analyzing TSS-Seq data. Strict filtering of snoRNA-related, and perhaps also other small RNA-related, TSS-Seq peaks should be performed prior to analysis and is required in order to draw conclusions.

## Transcription start sites at ends of protein-coding genes do not explain stress-induced DoGs

**Fig. 2:**
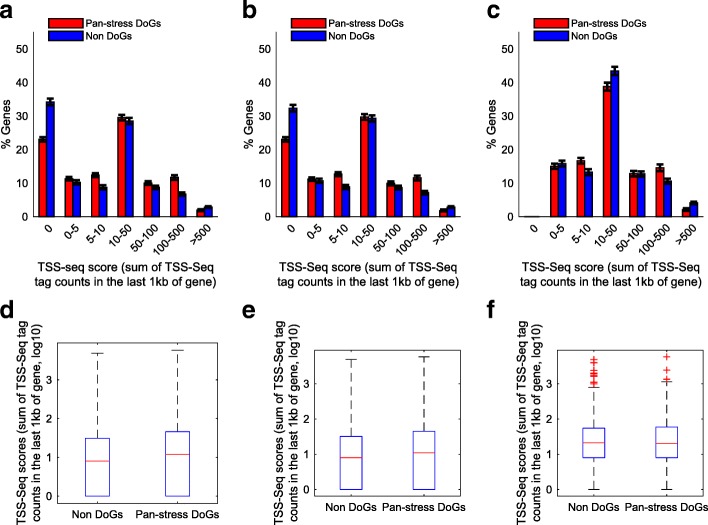
TSS-Seq peaks do not explain DoGs. TSS-Seq data from NIH3T3 cells were downloaded from DBTSS [8], and TSS-Seq peaks in the last 1kb of all genes were extracted using bedtools. Bar graphs **a**–**c** show the mean (and standard deviation) percentage of genes in each bin of TSS-Seq scores (sum of tag counts in the last 1kb) as calculated from 1000 sub-samples of expression-matched pan-stress DoG-associated and non-DoG-associated genes. Boxplots **d**–**f** show the cumulative distribution of TSS-Seq peak scores in log10 scale according to three inclusion criteria: **a**, **d** No filter: median TSS-Seq scores are 11 for pan-stress DoGs and 8 for non-DoG genes, *p* = 0.009. **b**, **d** Excluding 113 genes that harbor either a snoRNA or a TSS of another transcript within their last 1kb: median TSS-Seq score is 11 for pan-stress DoGs and 8 for non-DoG genes, *p* = 0.048. **c**, **e** Excluding additional 545 genes with zero TSS-Seq tag count in their last 1kb: median TSS-Seq score is 20 for pan-stress DoG- and 21 for non-DoG-associated genes, *p* = 0.37

Next, Huang and Liu compared the TSS-Seq tag counts in the last 1kb of pan-stress DoG-generating genes versus non-DoG genes and reported significant differences. We replicated the same analysis, while using stringent inclusion criteria for non-DoGs—genes that show no evidence of transcriptional readthrough. As in our previous study [6], we defined pan-stress DoGs as DoGs that exist in all three stress conditions, heat shock, oxidative, and osmotic stress. We further defined non-DoGs as the group of genes whose maximum reads per kilobase per million mapped reads (RPKM) over the 4kb region downstream of the gene end was lower than the minimal RPKM of the 4kb region downstream of the gene end of pan-stress DoGs, in all three stress conditions in our NIH3T3 RNA-seq dataset [6]. We analyzed the same TSS-Seq data, while performing rigorous multiple sub-sampling and expression-matching procedures, to generate 1000 matching pan-stress DoG and non-DoG sets, in order to ensure similar distributions of expression levels of their upstream associated genes (explained in [6]). We then calculated the median TSS tag counts in the last 1kb of the gene for both the pan-stress DoG and non-DoG groups. We found that the difference is significant (*p* = 0.009), but small: 11 for pan-stress DoGs and 8 for non-DoG genes (mean values are 65.8 and 62.9 for pan-stress DoGs and non-DoG genes, respectively. Fig. 2a, d). We then excluded 113 genes that harbor either a small RNA, e.g., a snoRNA or a TSS of another annotated transcript within their last 1kb, and repeated the same analysis. We found that although the median values remained identical, the difference now was only marginally significant (*p* = 0.048, mean values is now lower for pan-stress DoGs, 57.1, and 63.8 for non-DoG genes. Fig. 2b, e).

While examining the distributions of TSS-Seq tag counts in the last 1kb of pan-stress DoG- and non-DoG-associated genes (Fig. 2a, b), we noticed that the difference between them is mainly due to the number of genes with zero TSS-Seq tags. Indeed, when we excluded 545 additional genes that had zero TSS-Seq tag counts in their last 1kb, and repeated the analysis, the difference between pan-stress DoG- and non-DoG-associated genes completely disappeared, with median values of 20 and 21 for pan-stress DoG- and non-DoG-associated genes, respectively (*p* = 0.37, mean values are 70.6 and 94.1 for pan-stress DoGs and non-DoG genes, respectively. Fig. 2c, f). Thus, the minute difference between the TSS-Seq tag counts in the last 1 kb of pan-stress DoG- and non-DoG-associated genes that was originally observed in the NIH3T3 cell TSS-Seq data was driven by snoRNAs and true TSSs that should have been filtered, in addition to genes with zero TSS-Seq tag counts.

**Fig. 3:**
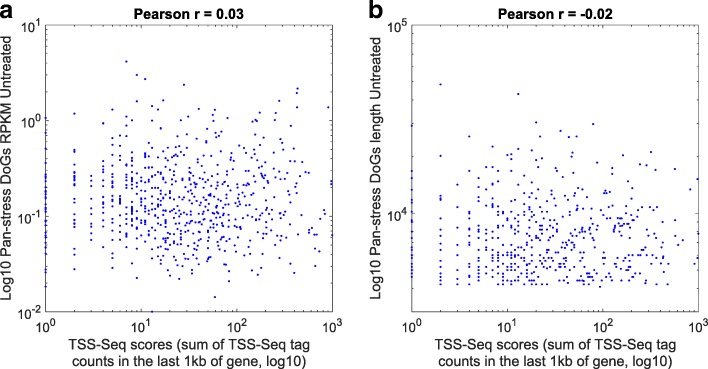
TSS-Seq peaks do not explain DoG expression and length. **a** Scatter plot of the RPKMs of all pan-stress DoGs (in log10 scale, from untreated cell RNAseq data [6]) versus the TSS-Seq scores in log10 scale; they show no correlation. **b** DoG lengths in unstressed cells [6] (in log10 scale) are plotted versus the TSS-Seq scores for each pan-stress DoG; here too, the correlation is close to zero. Pearson correlation coefficients are indicated

Moreover,  if DoGs are in fact generated by independent transcription, then TSS-Seq levels should correspond to DoG levels. However, we observe no correlation between TSS-Seq scores (TSS-Seq tag counts in the last 1kb of the gene) and DoG levels or DoG lengths (Pearson correlation of 0.03 and − 0.02 respectively, Fig. 3).

Finally, if independent transcription initiation were to produce DoGs, we should see significant increases in TSSs near the ends of all DoG-associated genes after stress. Huang and Liu analyzed data from unstressed cells only. Our previous Cap-Seq experiments, however, addressed this exact question by assaying stress-induced TSSs genome-wide, in human cells before and after osmotic stress [1]. Our results detected a reduction, not an increase, in Cap-Seq peak induction near the 3′-ends of DoG-associated human genes in osmotic stress compared to untreated cells [1]. Thus, we ruled out that osmotic-stress DoGs result from independent transcription initiation [1]. While future experiments after heat shock and oxidative stress should generalize this conclusion, the great overlap in the identity of DoG-producing genes between stress conditions that we previously reported [6] argues that, during stress, DoGs are generated by transcriptional readthrough.

## Abbreviations

DoG: Downstream of gene-containing transcript
lncRNA: Long non-coding RNA
RPKM: Reads per kilobase per million mapped reads
snoRNA: Small nucleolar RNA
TSS: Transcription start site
